# Population size, center–periphery, and seed dispersers’ effects on the genetic diversity and population structure of the Mediterranean relict shrub *Cneorum tricoccon*

**DOI:** 10.1002/ece3.2940

**Published:** 2017-08-05

**Authors:** Ana Lázaro‐Nogal, Silvia Matesanz, Alfredo García‐Fernández, Anna Traveset, Fernando Valladares

**Affiliations:** ^1^ Laboratorio Internacional de Cambio Global (LINC Global) Museo Nacional de Ciencias Naturales, MNCN‐CSIC Madrid Spain; ^2^ Área de Biodiversidad y Conservación ESCET Universidad Rey Juan Carlos Móstoles Spain; ^3^ LINC Global Institut Mediterrani d'Estudis Avancats, IMEDEA‐CSIC Esporles Mallorca Spain

**Keywords:** genetic diversity, islands, marginal populations, population size, relict plants, seed dispersal

## Abstract

The effect of population size on population genetic diversity and structure has rarely been studied jointly with other factors such as the position of a population within the species’ distribution range or the presence of mutualistic partners influencing dispersal. Understanding these determining factors for genetic variation is critical for conservation of relict plants that are generally suffering from genetic deterioration. Working with 16 populations of the vulnerable relict shrub *Cneorum tricoccon* throughout the majority of its western Mediterranean distribution range, and using nine polymorphic microsatellite markers, we examined the effects of periphery (peripheral vs. central), population size (large vs. small), and seed disperser (introduced carnivores vs. endemic lizards) on the genetic diversity and population structure of the species. Contrasting genetic variation (*H*_E_: 0.04–0.476) was found across populations. Peripheral populations showed lower genetic diversity, but this was dependent on population size. Large peripheral populations showed high levels of genetic diversity, whereas small central populations were less diverse. Significant isolation by distance was detected, indicating that the effect of long‐distance gene flow is limited relative to that of genetic drift, probably due to high selfing rates (*F*_IS_ = 0.155–0.887), restricted pollen flow, and ineffective seed dispersal. Bayesian clustering also supported the strong population differentiation and highly fragmented structure. Contrary to expectations, the type of disperser showed no significant effect on either population genetic diversity or structure. Our results challenge the idea of an effect of periphery *per se* that can be mainly explained by population size, drawing attention to the need of integrative approaches considering different determinants of genetic variation. Furthermore, the very low genetic diversity observed in several small populations and the strong among‐population differentiation highlight the conservation value of large populations throughout the species’ range, particularly in light of climate change and direct human threats.

## 1 Introduction

Relict species are generally expected to show low levels of genetic diversity and strong population structure (Awad, Fady, Khater, Roig, & Cheddadi, 2014; Bauert, Kalin, Baltisberger, & Edwards, 1998; Ge et al., 2005) because they are usually composed of small, geographically isolated populations (Dobrowski, 2011; Hampe & Petit, 2005). However, experimental evidence has not always supported this generalization (see Peakall, Ebert, Scott, Meagher, & Offord, 2003; Vanden‐Broeck et al., 2011). Low rates of evolution, genetic stability, and habitat reliability have been proposed as some of the factors that may influence the genetic patterns of relict species (Hampe & Petit, 2007). Information on genetic diversity and population structure—and their determinant factors—along a species’ distribution range has shown to be critical for designing conservation strategies for endangered relict species (Lesica & Allendorf, 1995).

Differences in genetic diversity and structure between peripheral and central populations have long been studied (e.g., Arnaud‐Haond et al., 2006; Durka, 1999; Eckert, Samis, & Lougheed, 2008; Eckstein, O'neill, Danihelka, Otte, & Köhler, 2006; Lammi, Siikamäki, & Mustajärvi, 1999; Pironon et al., 2016; Van Rossum, Vekemans, Gratia, & Meerts, 2003), but a clear pattern has not yet been found. It is often assumed that peripheral populations are small, isolated, and occur in ecologically marginal habitats where selection pressures are likely to be more intense (Brown, Stevens, & Kaufman, 1996; Eckert et al., 2008; Lawton, 1993; Lesica & Allendorf, 1995; Pulliam, 2000). Such populations can have low genetic diversity as a consequence of high inbreeding, genetic drift, and directional selection and may also show strong genetic structure due to reduced gene flow (Arnaud‐Haond et al., 2006; Durka, 1999; Gapare & Aitken, 2005; Lammi et al., 1999; Schaal & Leverich, 1996). However, it is not known to what extent the effects of periphery are confounded by those of population size. Populations established in peripheral areas can be smaller, equally large (see e.g., Dolan, 1994; Van Rossum et al., 2003) or even larger than central ones (see e.g., Kluth & Bruelheide, 2005), which could modulate the effect of periphery. Therefore, genetic diversity of peripheral populations could be expected to be similar to that of central ones. Accordingly, central distributions with small populations might have lower genetic diversity. Despite these implications, to our knowledge, the effects of population size on genetic diversity have been rarely studied in conjunction with other key factors, such as location within the species distribution range (i.e., center–periphery; see Pironon et al., 2016).

In the case of Mediterranean species, reproductive system, colonization success, and dispersal abilities have also been recognized as important aspects that could affect species’ evolution (Feliner, 2014) as well as shape their genetic diversity. For instance, species with long‐range seed dispersal mechanisms, such as zoochory, often have higher within‐population genetic diversity and lower population differentiation than species with limited dispersal (Avise, 2004; Hamrick, Murawski, & Nason, 1993; Vanden‐Broeck et al., 2011). Therefore, the loss of local vertebrate frugivores can have detrimental genetic effects on plants that depend upon them (Babweteera & Brown, 2009; Christian, 2001). Seed dispersal disruptions can impact seed removal success and regeneration (Riera, Traveset, & García, 2002; Traveset, 1995b; Traveset, Gonzalez‐Varo, & Valido, 2012) and may lead to the loss of genetic variation and inbreeding due to increased drift and clumping (Jordano et al., 2011). However, the genetic consequences of seed disperser loss have so far been poorly investigated (but see Calviño‐Cancela et al., 2012; Pérez‐Méndez, Jordano, & Valido, 2015), particularly in the face of other key aspects such as population size and center–periphery effects.

The Mediterranean basin is an important area for relict species, including those that emerged during the Tertiary (Palamarev, 1989). Many of these Tertiary relicts have evolved key traits (e.g., long life cycles, sprouting, seed bank development, and fleshy fruits for seed dispersion) to survive and enhance their performance (see revision in Rodríguez‐Sánchez, Perez‐Barrales, Ojeda, Vargas, & Arroyo, 2008). These adaptations, together with the climate oscillations during the Quaternary, the changes in sea level, and the tectonic movements around the Mediterranean Sea are some of the processes that have governed the complexity of the Mediterranean flora, making it a biodiversity hot spot (Myers, Mittermeier, Mittermeier, Da Fonseca, & Kent, 2000). However, these processes also complicate the formation of common patterns that could explain the biogeographic distributions of Mediterranean species and the test of general hypotheses (Feliner, 2014), such as the center (abundant)–periphery (marginal) rule (Pironon et al., 2016; Sagarin & Gaines, 2002).

**Figure 1:**
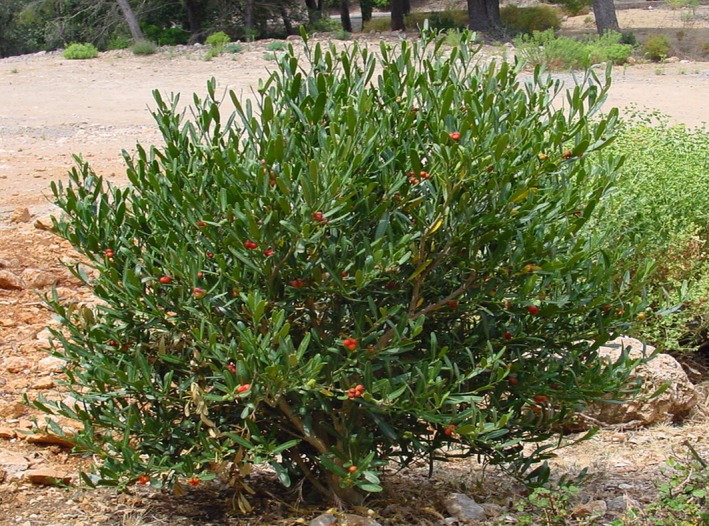
Reproductive individual of the study species, the relict shrub *Cneorum tricoccon*

*Cneorum tricoccon* L. (Figure 1) is a Mediterranean relict shrub of limited and regressive distribution (Lázaro‐Nogal, Forner, Traveset, & Valladares, 2013; Traveset, 1995a). It is endemic to the western Mediterranean area, the Balearic Islands being their main distribution area. Isolated peripheral populations are found in the Iberian Peninsula, France, Sardinia, and Tuscany. *Cneorum tricoccon* has a tight seed dispersal mutualism with endemic lizards, but the introduction of carnivorous mammals in the Balearic Islands contributed to their extinction (particularly from the islands of Mallorca and Menorca) and, consequently, disrupted seed dispersal in this and other systems (Riera et al., 2002; Traveset, 1995b). Currently, in populations where lizards are extinct, some of those carnivorous mammals (mainly pine martens *Martes martes* L.) replace them to some extent as seed dispersers (Celedón‐Neghme, Traveset, & Calviño‐Cancela, 2013), but its consequences for the population genetics of *C. triccocon* are still unknown.

Our aim in this study was to assess genetic diversity and structure of *C. tricoccon* populations encompassing the entire distribution range of the plant, using microsatellite markers. We hypothesized that there will be differences in genetic diversity between populations of similar size located in the center versus the periphery of the species range. Moreover, we predicted that populations where native dispersers are missing would also show different genetic patterns than those in which native dispersers still exist. We selected a set of 16 populations of contrasting population size, periphery, and type of seed disperser. Specifically, we addressed the following questions: (1) Are there differences in genetic diversity and structure among populations in relation to the population's position in the distribution range (i.e., center vs. periphery) or population size (large vs. small)? (2) Are introduced mammals effective dispersers promoting greater genetic and structural diversity compared to the native lizards?

## 2 Methods

### 2.1 Study species and population sampling

*Cneorum tricoccon* L. (Cneoraceae) is a perennial shrub approximately 1 m tall, although some individuals can reach up to 2 m. It is an andromonoecious, insect‐pollinated, and self‐compatible species (Traveset, 1995a). It represents a relict species of the Tertiary, which evolved under tropical climate conditions (Raven, 1973). It has been categorized as vulnerable (IUCN, International Union for Conservation of Nature) with human activities and climate change the major threats to the species’ survival. Its distribution has diminished in recent decades with some local extinctions (Traveset, Quintana, & Alcover, 2005). It inhabits the western Mediterranean area, with populations in the Balearic Islands considered the center of the species distribution range, and populations in the eastern and southern Iberian Peninsula, southeastern France, Sardinia, Giannutri, and Tuscany considered peripheral populations (Traveset, 1995a). In the Balearic Islands, seed dispersal is mostly mediated by endemic lizards (*Podarcis lilfordi*,* P. pityusensis,* and *P. siculus*). However, introduced carnivorous mammals (approximately 250 B.C.), such as pine martens (*M. martes)* or genets (*Genetta genetta* L.), are currently the main dispersers in localities where lizards are extinct (mainly Mallorca and Menorca; Traveset, 1995b; Celedón‐Neghme et al., 2013).

**Figure 2:**
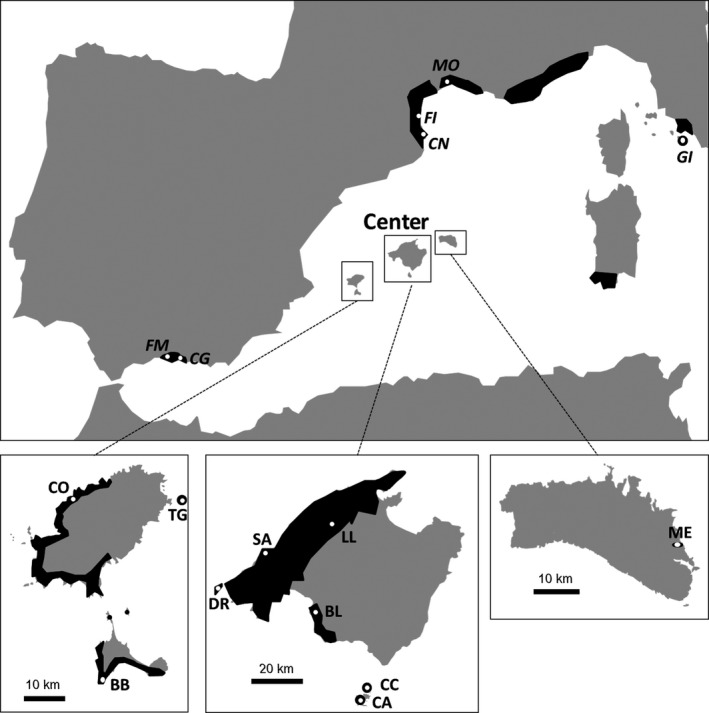
Distribution of *Cneorum tricoccon* (black areas) and location (white dots) of the populations sampled. Letter codes correspond to those listed in Table 1. Codes in italics refer to peripheral populations

A total of 399 individuals were sampled in 16 populations (Table 1, Figure 2) spanning the entire species’ distribution range. Selected populations covered contrasting levels of periphery (central vs. peripheral) and population size (large vs. small), and they differed in their main disperser (endemic lizards vs. introduced carnivorous mammals). To distinguish among peripheral and central populations, we followed a geographic selection criterion (Lesica & Allendorf, 1995; Pironon et al., 2016). We considered central populations as thriving populations located in the main species distribution (i.e., Balearic Islands) and peripheral as the other scant populations that appear in the mainland (Mediterranean coasts, Figure 2). We extensively sampled the extant populations located outside the main distribution area, and were able to include six of the seven extant peripheral populations in this study. To define the two levels of population size, we considered small populations those with <200 individuals and large populations those with >200 individuals (Frankham, 1996; Frankham, Briscoe, & Ballou, 2002), a relatively conservative threshold used in several studies (see e.g., Kery, Matthies, & Spillmann, 2000). Previous available field data and information on the species dispersal support our categorization (A. Traveset, personal communication). Complete information about the disperser type present in each population is available only for populations in the Balearic Islands. Consequently, we have tested the effect of this factor in a subsample of all populations, using a classification based on previous available data (Pérez‐Mellado et al., 2008; Traveset, 1995b). Although there has not been any specific study on the dispersers in the peripheral populations, we know from personal observations and communications that carnivores, such as pine and stone martens, are the main frugivores in the mainland. Carnivore scats were occasionally found with *C. tricoccon* seeds in the Iberian Peninsula populations, and other studies have shown the importance of carnivores as seed dispersers for a large variety of fleshy fruited species (e.g., González‐Varo, López‐Bao, & Guitián, 2013).

**Table 1:** Population code, location, and characterization of the 16 sampled *Cneorum tricoccon* populations

Code	Population	Region	Lat	Long	Insularity	Pop Size	Disperser	Habitat
Central populations								
SA	S'Arboçar	Mallorca	39.678	2.544	LIs	L	Mammals	Oak forest
LL	Lluc	Mallorca	39.789	2.865	LIs	L	Mammals	Oak forest
BL	Cap Blanc	Mallorca	39.479	2.738	LIs	L	Mammals	Maquis
CO	Pla de Corona	Ibiza	39.044	1.333	SIs	L	Lizards	Maquis
TG	Tagomago	Ibiza	39.035	1.644	SIs	L	Lizards	Maquis
BB	Cap de Barbaria	Formentera	38.642	1.39	SIs	L	Lizards	Maquis
CA	Cabrera	Cabrera	39.150	2.953	SIs	S	Lizards	Maquis
CC	Conillera de Cabrera	Cabrera	39.182	2.962	SIs	S	Lizards	Maquis
DR	Dragonera	Dragonera	39.587	2.329	SIs	L	Lizards	Maquis
ME	Sa Mesquida	Menorca	39.917	4.287	LIs	S	Mammals	Maquis
Peripheral populations								
GI	Gianuttri	Italy	42.255	11.099	SIs	L	?	Maquis
CR	Cap de Creus	NE Spain	42.322	3.318	Con	S	?	Maquis
FI	Fitou	France	43.54	3.785	Con	L	?	Maquis
MO	Montpellier	France	42.892	2.978	Con	L	?	Maquis
CG	Cerro Gordo	S Spain	36.741	−3.777	Con	S	?	Maquis
MA	Fuente de Maro	S Spain	36.759	−.85	Con	S	?	Maquis

Lat, latitude; Long, longitude; LIs, large island; SIs, small island; Con, continent; L, large (>200); S, small (<200);?, incomplete information on dispersers.

We sampled 21–30 indiscriminately selected individuals within areas of about 1 km^2^ per population. Leaves for DNA extraction (2–3) were collected and stored in bags with silica gel.

### 2.2 DNA extraction, microsatellite analysis, and fragment scoring

Total genomic DNA was extracted from 20 mg of dry leaf tissue using the DNeasy Plant Mini Kit (Qiagen, Valencia, CA, USA) following the manufacturer's protocol. Its concentration and purity were quantified on a NanoDrop spectrophotometer (NanoDrop Products, Wilmington, DE, USA).

We genotyped each sample at nine microsatellite loci known to be polymorphic across the species, described in Garcia‐Fernandez, Lazaro‐Nogal, Traveset, and Valladares (2012): Ctric 00490, Ctric 02925, Ctric 07615, Ctric 14301, Ctric 06384, Ctric 10195, Ctric 19884, Ctric 15341, and Ctric 09344. An M13 tail was added to one of the PCR primers (Schuelke, 2000). PCRs were performed in a 10 μl mix containing 4.1 μl of RNase‐free water, 1× Taq buffer (Biotools, Madrid, Spain), 2 mmol/L MgCl_2_, 0.25 μmol/L of each dNTP, 0.04 μmol/L of the forward primer with M13 tail, 0.16 μmol/L of the reverse primer and 0.16 μmol/L of the universal fluorescently labeled M13 primer (6‐FAM, VIC, PET, and NED), 0.5 U of Taq polymerase (Biotools), and 10 ng of template DNA. The PCR program consisted of one step of 4 min at 94°C followed by 30 cycles each of 30 s at 95°C, 45 s at 56°C, and 45 s at 72°C. Another eight cycles were then performed, consisting of 30 s at 95°C, 45 s at 53°C, and 45 s at 72°C, followed by a final step of 10 min at 72°C. A S1000 Thermal Cycler (Bio‐Rad laboratories, Richmond, CA, USA) was used. Each reaction was checked for successful amplification by running 3 μl of PCR product of six indiscriminately selected individuals per population and microsatellite locus on a 1% agarose gel stained with SYBR Safe (Invitrogen, Carlsbad, CA, USA). PCR products (1 μl) were diluted with 9.1 μl of a loading mixture containing 9 μl of HiDi Formamide and 0.1 μl GeneScan LIZ500 internal size standard (Applied Biosystems, Foster City, CA, USA) and analyzed on an automated DNA sequencer (ABI PRISM 3730 Genetic Analyser, Applied Biosystems) in Unidad de Genómica, Parque Científico de Madrid, Spain. Amplified fragment lengths were scored with GENEMARKER v. 2.4.0 (Softgenetics LLC, State College, PA, USA).

### 2.3 Data analysis

#### 2.3.1 Genetic diversity within populations

Genetic diversity indices, including *P*, percentage of polymorphic loci; *A*, mean number of alleles per locus (allelic richness); *A*_E_, mean number of effective alleles ($1/\Sigma p_{i}^{2}$, where *p*_*i*_ is the frequency of the *i*th allele for the population); *H*_O,_ observed heterozygosity (number of heterozygotes/*N*, where *N* is the number of individuals per population); *H*_E,_ expected heterozygosity ($1-\Sigma p_{i}^{2}$); *F*_IS_, fixation index (1 − (*H*_O_/*H*_E_)), and the number of private alleles, were estimated using GENALEX v. 6.41 (Peakall & Smouse, 2006). *F*_IS_ was estimated using INEst 2.0 software (Chybicki & Burczyk, 2009) that corrected for the excess of homozygosity due to the effects of null alleles and genotyping errors (50 × 10^5^ Markov chain Monte Carlo [MCMC] iterations, burn‐in = 50,000 and thinning = 50). Deviation from the Hardy–Weinberg equilibrium (HW) was evaluated at each population with the MCMC approximation (dememorization = 10,000, batches = 100, iterations per batch = 10,000) of Fischer's test implemented in GENEPOP V. 4.1 (Rousset, 2008). The same software was used to check for genotypic linkage disequilibrium (LD) between pairs of loci within each population using the log‐likelihood ratio G statistic (dememorization = 10,000, batches = 100, iterations per batch = 10,000).

#### 2.3.2 Population structure and differentiation

To determine population differentiation, we calculated pairwise *F*_ST_ values (Weir & Cockerman, 1984) using FreeNA, which implements a correction to provide accurate estimation of *F*_ST_ in the presence of null alleles (Chapuis & Estoup, 2007). A Mantel test (Legendre & Legendre, 1998) was performed to check for the correlation between genetic and geographical distances among populations (isolation by distance [IBD]). The matrix of genetic differentiation was calculated with pairwise *F*_ST_ values, and the matrix of geographical (Euclidean) distances was generated with PASSAGE v. 2 (Rosenberg & Anderson, 2011). The Mantel test was carried out also with the PASSAGE software, using 10,000 permutations. This was repeated using the matrix of logarithm transformation of the distance among populations, which could normalize the distribution.

Population genetic structure in central and peripheral regions was examined by means of analysis of molecular variance (AMOVA, Excoffier, Smouse, & Quattro, 1992) computed in ARLEQUIN. We examined the distribution of genetic variation at three hierarchical levels: (1) among regions (center vs. periphery), (2) among populations within regions, and (3) within populations. We thus computed three fixation indices: *F*_CT_ (variation among regions), *F*_SC_ (among populations within regions), and *F*_ST_ (among all populations). The significance of the variance components was tested using nonparametric permutation procedures, with 50,000 permutations.

Genetic structure was also analyzed using Bayesian clustering methods implemented in STRUCTURE v. 2.3.1. (Pritchard, Stephens, & Donnelly, 2000), comparing the results obtained with AMOVA with a different statistical approach. STRUCTURE assumes a model in which there are *K* genetic clusters (where *K* is unknown), where each *K* is characterized by a set of allele frequencies at each locus. Individuals are probabilistically assigned to one or more clusters. Our analyses were based on an admixture ancestral model with correlated allele frequencies (Falush, Stephens, & Pritchard, 2003) for a range of *K* values starting from two to the number of populations plus two (i.e., 18). The proportion of membership of each individual and population to the inferred *K* cluster was then calculated. We performed 12 independent runs for each *K,* using a burn‐in period and a run length for the MCMC of 5 × 10^5^ and 10^6^ iterations, respectively. We used HARVESTER (Earl & vonHoldt, 2011) to extract the relevant data from STRUCTURE and to determine the number of clusters most appropriate for the interpretation of our data. First, HARVESTER calculates the mean log probability of the data for each *K*. Second, it calculates ∆*K* following the method described in Evanno, Regnaut, and Goudet (2005). ∆*K* is an ad hoc parameter which estimates the rate of change in the log probability of the data between the successive *K* values. We then used CLUMPP v. 1.2.2 (Jacobsson & Rosenberg, 2007) to combine results from the 12 runs at each *K*, using the *Greedy* option (for *K *<* *9) and the *LargeKGreedy* option (for *K *≥* *10). Membership in clusters was graphically represented using DISTRUCT v. 1.1 (Rosenberg, 2004). Additionally, a Bayesian analysis was conducted using the R package GENELAND (Guillot, Mortier, & Estoup, 2005), which also uses MCMC algorithms to perform clustering analyses with the option to include nonspatial as well as spatial models. In this case, we performed spatial clustering; MCMC iterations were set at 10^5^, thinning at 100, and the burn‐in period of 100. The number of *K* to be tested was set at 1–18, and five replicates for each possible *K* were run. The best result was chosen, based on the highest average posterior probability. Finally, a principal coordinates analysis (PCoA) based on *F*_ST_ pairwise values was performed in GENALEX to provide further insight into population differences.

#### 2.3.3 Effects of center–periphery, population size, and disperser type on genetic variation and population structure

To assess the effects of the study factors on within‐population genetic diversity and population structure, we compared diversity indices (*A*,* H*_O_, *H*_S_, *F*_IS_) and differentiation (*F*_ST_) among groups using FSTAT v. 2.9.3 3 (Goudet, 1995). Populations were grouped (see Table 1) according to periphery (central vs. peripheral), population size (large vs. small), and disperser type (lizard vs. mammal), and significance was assessed based on 10,000 permutations.

## 3 Results

### 3.1 Genetic diversity within populations and comparisons between populations

A total of 80 alleles were scored from the nine microsatellites in the 399 individuals analyzed, an average of 8.89 alleles per locus. The number of alleles per locus ranged from 6 to 16. Genetic diversity varied significantly across populations (summarized data for genetic diversity indices are shown in Table 2). These differences were not related to population location or disperser type; that is, there were no significant differences in the genetic diversity indices when peripheral versus central populations were compared or when populations were grouped according to disperser type (endemic lizards vs. introduced mammals) (Table 3). Conversely, we found an effect of population size on genetic diversity: *A*,* H*_O_, and *H*_S_ were lower and *F*_ST_ was larger in small compared to large populations (Table 3).

**Table 2:** Genetic diversity indices of the 16 *Cneorum tricoccon* populations, using nine microsatellite loci

Population code	*N*	*P*	*A*	*A*_E_	*H*_O_	*H*_E_	*F*_IS_	Private alleles
Central populations								
SA	30	89	4.11	2.82	0.366	0.476	0.155^a^	1
LL	25	100	3.67	2.33	0.182	0.485	0.651	3
BL	25	100	3.89	2.21	0.142	0.477	0.731^a^	0
CO	25	100	3.78	2.21	0.273	0.482	0.476^a^	1
TG	25	89	3.33	1.95	0.178	0.387	0.550^a^	0
BB	25	100	2.89	1.8	0.089	0.408	0.786^a^	2
CA	25	11	1.11	1	0.004	0.004	0.887 (8)^a^	0
CC	25	67	2.11	1.83	0.209	0.354	0.453 (3)^a^	0
DR	25	100	2.89	1.66	0.173	0.366	0.555^a^	5
ME	23	11	1.11	1.04	0.039	0.032	0.865 (8)^a^	0
Peripheral populations								
GI	21	56	1.89	1.22	0.027	0.132	0.795 (4)^a^	3
CR	25	44	1.56	1.27	0.032	0.144	0.780 (5)^a^	1
FI	25	100	3.56	1.97	0.236	0.437	0.481^a^	3
MO	25	56	1.89	1.39	0.102	0.207	0.540 (4)^a^	0
MA	25	33	1.67	1.12	0.036	0.079	0.591 (6)^a^	1
CG	25	56	1.78	1.14	0.04	0.085	0.556 (4)^a^	1

*N*, number of individuals sampled; *P*, proportion of polymorphic loci; *A*, mean number of alleles per locus (allelic richness); *A*_E_, mean number of effective alleles, *H*_O_, observed heterozygosity; *H*_E_, expected heterozygosity; *F*_IS_ inbreeding coefficient and number of private alleles. The number of monomorphic loci is shown in parentheses.

a Significant departures from Hardy–Weinberg equilibrium (*P *≤* *0.001). See text for details on statistics. Letter codes are listed in Table 1.

**Table 3:** Effects of center–periphery, population size, and disperser type on genetic diversity and population structure of *Cneorum tricoccon*

	Center–Periphery			Population size			Disperser		
	Central	Peripheral	*p*	Large	Small	*p*	Lizard	Mammal	*p*
*A*	2.672	1.940	.113	2.933	1.505	**.001**	2.549	2.855	.613
*H*_O_	0.169	0.080	.113	0.182	0.06	**.002**	0.152	0.192	.595
*H*_E_	0.362	0.188	.060†	0.403	0.121	**.001**	0.342	0.389	.705
*F*_IS_	0.533	0.575	.740	0.550	0.504	.713	0.555	0.506	.732
*F*_ST_	0.567	0.725	.106	0.457	0.822	**.004**	0.527	0.488	.827

Grouping comparisons were tested for significance using 10,000 permutations. Bold figures are significant (*p *<* *.01) and ^†^ are marginally significant.

The percentage of polymorphic loci (*P*) was high in central populations with the exception of CA and ME, in which 89% of the loci were monomorphic. Conversely, *P* was low (33%–56%) in all periphery populations, except in FI, where all loci were polymorphic. The lowest mean number of alleles per locus (*A*) was found in CA and ME (1.11), whereas SA had the largest value (4.11). The mean number of effective alleles (*A*_E_) showed similar results, ranging from 1.0 (in CA) to 2.82 (in SA). Observed (*H*_O_) and expected (*H*_E_) heterozygosities tended to be higher in central populations than in peripheral populations (with the exception of CA, ME, and FI), but differences were not significant (Table 3). Observed heterozygosities (*H*_O_) ranged from 0.004 (CA) to 0.366 (SA), whereas expected heterozygosities (*H*_E_) ranged from 0.004 (CA) to 0.485 (LL). The inbreeding coefficient (*F*_IS_) varied among populations from 0.185 (SA) to 0.887 (CA) and also showed a (not‐significant) tendency to be higher in peripheral populations, with the exception of CA and ME, which showed the highest *F*_IS_ values. We found a total of 20 private alleles, present in 10 of the 16 populations. The number of private alleles per population ranged from one (SA, CO, CR, MA, CG) to five (DR). All populations showed significant departures from HW equilibrium toward heterozygote deficiency (*p *<* *.001 across loci and populations). No consistent LD was found between any pairwise comparisons across loci and populations (data not shown).

### 3.2 Genetic structure and population differentiation

All pairwise *F*_ST_ values of differentiation among populations were highly significant and generally very high (Table S1), ranging from 0.153, between MA and CG, to 0.977 between CA and ME, with an overall *F*_ST_ of 0.556 ± 0.179 (mean ± SD). Pairwise *F*_ST_ values were higher than 0.5 in ≥70% of the comparisons. In general, *F*_ST_ values were lower between nearby populations (e.g., MA and CG). Accordingly, we detected significant IBD between populations, either using the matrix of linear Euclidean distances (*r*_M_ = 0.34; *p *=* *.039) or the logarithm of the distances (*r*_M_ = 0.37; *p *=* *.008; Fig. S1). Hierarchical AMOVA (Table 4) showed a high proportion of genetic variation among (57.3%) and within populations (40.1%), while a very small fraction of the variation was due to differences among regions (central vs. peripheral) (2.52%).

**Table 4:** Population genetic structure inferred by hierarchical analysis of molecular variance and Weir and Cockerham’ *F*‐statistics estimates: *F*_CT_, variation among regions (peripheral vs. central populations); *F*_SC_, among populations within regions; and *F*_ST_, among all populations

Source of variation	*df*	Sum squares	Variance components	% Variation
Among regions (central vs. peripheral)	1	124.115	0.082	2.52 ns
Among populations within regions	14	1,321.098	1.866	57.34***
Within populations	782	1,021.394	1.306	40.14***

Fixation indices		*p*‐Value		
*F*_ST_	0.599	***		
*F*_SC_	0.588	***		
*F*_CT_	0.025	.127		

The significance of the variance components was tested using nonparametric permutation procedures with 50,000 permutations. ***, *P*<0.001

**Figure 3:**
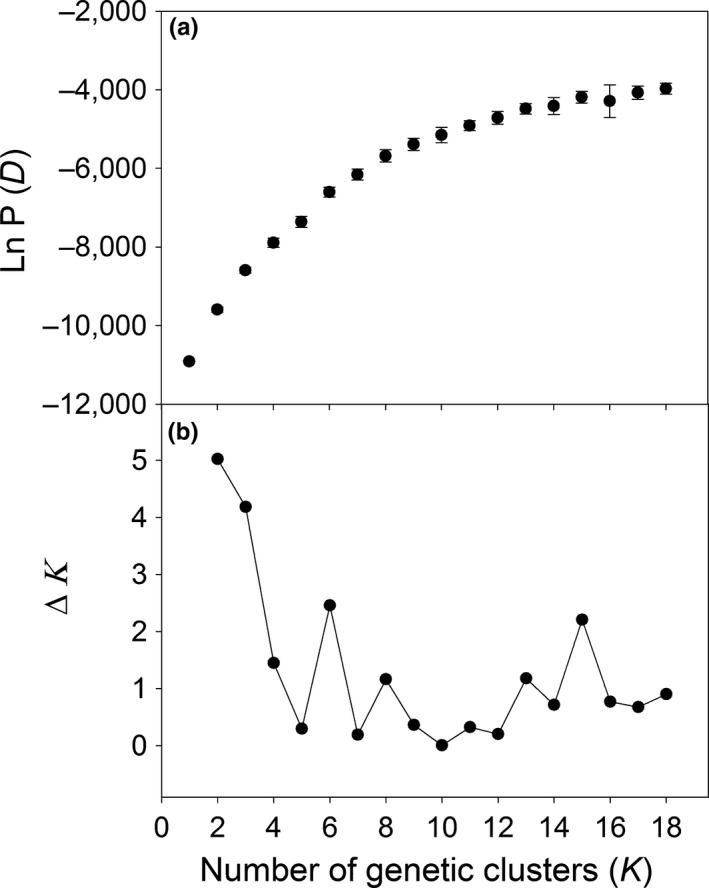
(a) Number of genetic clusters (*K*) detected by Evanno et al. (2005). Mean log probability of the data for the 12 STRUCTURE runs at each *K*. Error bars are SD; (b) Δ*K*, rate of change in the log probability of data between successive *K* values

**Figure 4:**
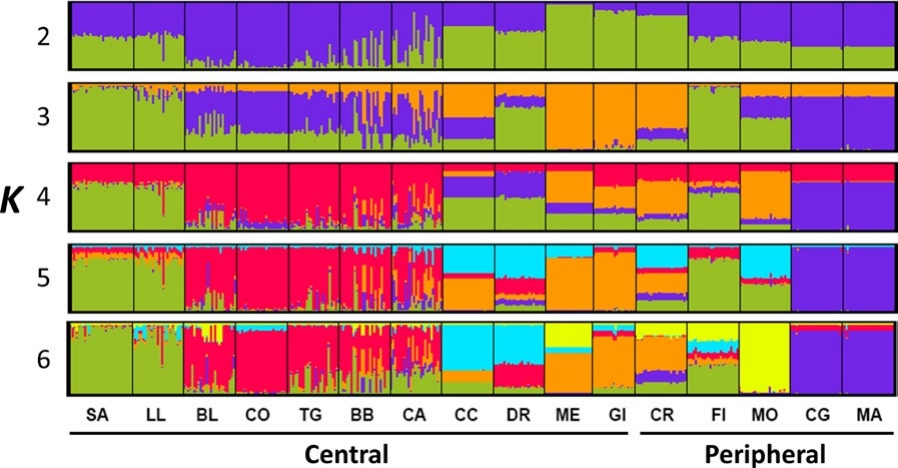
Population structure inferred by Bayesian cluster analyses (STRUCTURE) for 399 *Cneorum tricoccon* individuals from 16 central and peripheral populations. Results for *K* (number of clusters) ranging from 2 to 6. Each individual (grouped by population) is represented by a vertical bar. Letter codes correspond to the population listed in Table 1

Results from Bayesian clustering conducted by STRUCTURE and strictly interpreted using the method of Evanno et al., 2005 would suggest that two genetic clusters are sufficient for interpretation of our data (*K *=* *2, Figure 3). However, we focus on the results with *K *=* *6 and *K *=* *15 because there are two secondary large peaks in ∆*K* at *K *=* *6 and *K *=* *15 that could explain a secondary substructure (Figure 3). Moreover, larger values of *K* are consistent with the observed high among‐population differentiation revealed by AMOVA and by high pairwise *F*_ST_ values. We thus show results for *K *=* *2‐6 to provide a comprehensive understanding of population structure (Figure 4). In the *K *=* *6 solution, all populations contained admixed individuals; that is, an individual was assigned to different genetic clusters (Figure 4). However, several populations were assigned to a predominant cluster; for example, SA and LL were included mainly in the green cluster; CO, TG, BB, DR, BL, and CA in the red one; CG and MA in the purple one. In general, nearby populations were assigned to the same cluster (e.g., SA and LL; CG and MA); however, there were also close populations included in different clusters (e.g., CA and CC; FI and MO). Central and peripheral populations were generally assigned to different clusters. In the *K *=* *15 solution, all populations were assigned to different clusters, except for the nearest populations (CG and MA) that were included in the same cluster (Fig. S2).

The clustering obtained with *K *=* *6 was in general consistent with results from a PCoA based on *F*_ST_ distances (Fig. S3). The PCoA also revealed the strong differentiation of CA and ME to other populations, which was only observed at larger *K* values in the Bayesian analysis. Furthermore, these results were comparable to the second Bayesian clustering method used, GENELAND, which allowed inclusion of the geographical location of the populations. Again, 15 clusters were found as the most plausible scenario for explaining the data structure. This approach assigned each population to different clusters, except for CG and FM that were grouped together, which clearly supports the observed high genetic structure.

## 4 Discussion

While several studies have shown reduced genetic diversity in peripheral compared with central populations (Arnaud‐Haond et al., 2006; Durka, 1999; Eckert et al., 2008; Eckstein et al., 2006; Lammi et al., 1999), our results showed an alternative pattern where other interacting factors may override the presumed effects of the center–periphery hypothesis. *Cneorum tricoccon* populations located at range margins tended to show lower genetic diversity (*P*,* A*,* H*_O,_ *H*_E_) and higher inbreeding (*F*_IS_) than central populations, although differences were not significant. In contrast, population size was significantly associated with most genetic diversity indices. It is generally predicted that central populations are larger than peripheral ones (Eckert et al., 2008; Lawton, 1993; Sagarin & Gaines, 2002). However, *C. tricoccon* contains several large populations at the range margins and small ones in the center. The large peripheral populations (e.g., FI) maintain high levels of genetic diversity, despite their geographic isolation. Similarly, small populations located in the center of the species distribution (e.g., CA, ME) are genetically depauperated, showing even lower genetic diversity than peripheral populations. Our results support the stronger role of population size in determining the genetic diversity of populations (Ellstrand & Elam, 1993) and, more importantly, its potential to counteract the effect of periphery. Furthermore, our results add insight into scenarios where the central–marginal hypothesis may not hold due to insularity and isolation effects (Pironon et al., 2016).

The several methods used to assess population structure showed high genetic differentiation among *C. tricoccon* populations. According to hierarchical AMOVA, 57% of the variation was found among populations, and mean *F*_ST_ value was 0.56, much higher than the average of *F*_ST_ = 0.24 for plant species with mixed breeding systems (Nybom, 2004). Concurrent with the strong population structure, we also found a pattern of IBD; that is, closer populations were genetically more similar than populations farther apart (see Awad et al., 2014; Eckstein et al., 2006). Spatial distribution of genetic variation in plants is the outcome of several factors such as mating system, gene flow, genetic drift, and natural selection (Petit & Hampe, 2006). Our results suggest that gene flow among populations is limited, likely due to a combination of different factors, such as high selfing rates, populations with high kinships coefficients between parents (Hirao, 2010), and limited pollen and seed dispersal. In general, outcrossing species maintain most genetic variation within populations, while selfing species harbor comparatively higher variation among populations (Avise, 2004). *Cneorum tricoccon* is a self‐compatible species, and, thus, its marked population structure and high inbreeding coefficients suggest that selfing (and/or crosses between parents with moderate–high kinship) could be more frequent than outcrossing in current conditions, likely due to the limited pollinator visits observed in many populations (A. Traveset, personal communication). Limited gene flow among populations is also in agreement with the strong genetic drift observed in certain populations (e.g., CA and ME), where many alleles were fixed. Finally, as has been documented in other long live Tertiary relicts, gene flow may have been interrupted during some historic periods, increasing isolation and therefore *F*_ST_ values (Dubreuil, Riba, & Mayol, 2008; Rodríguez‐Sánchez, Guzman, Valido, Vargas, & Arroyo, 2009; Rodríguez‐Sánchez et al., 2008).

As a Tertiary relict, *C. tricoccon* survived during several Quaternary climate oscillations and, as such, its phylogeographic history may also have altered its genetic patterns (Rodríguez‐Sánchez et al., 2008). The high level of genetic differentiation, the presence of a marked spatial structure, and the isolation‐by‐distance pattern found must be interpreted in the complex phylogeographic context of the Mediterranean basin (Feliner, 2014; Rodríguez‐Sánchez et al., 2008). *Cneorum tricoccon* has experienced marine transgression during the glacial and interglacial periods of the Quaternary, which greatly influenced the Balearic Islands (Vesica et al., 2000). Furthermore, the Messinian Salinity Crisis that almost desiccated the Mediterranean Sea, the recurrent connections, and disconnections with Northern Africa vegetation (Rodríguez‐Sánchez et al., 2008) or the existence of microrefugia for relictic species in the Mediterranean coasts (MolEco; Dubreuil et al., 2008; Lumaret et al., 2002) may have also influenced current species’ distributions (Hewitt, 2011). All these processes have fostered both expansion pulses—such as the several contacts of the Balearic Islands with the Iberian Peninsula (Garnatje, Pérez‐Collazos, Pellicer, & Catalán, 2013) or the cohesion of Mallorca and Cabrera in a single land mass (Vesica et al., 2000)—as well as isolation processes of the populations that colonized the extremes of the distribution (such as MA, CG, or GI).

Factors related to seed dispersal and insularity can also account for the high divergence observed. *Cneorum tricoccon* has a tight seed dispersal mutualism with lizards. The existence of natural dispersers may also be considered as a biological criterion to define optimal and marginal populations (Pironon et al., 2016). Lizards are known to be highly territorial and thus do not move seeds to long distances as do other vertebrates, such as birds or mammals. Furthermore, several *C. tricoccon* populations are restricted to small islands and islets and, consequently, are strongly isolated from each other. Past fragmentation leading to geographical isolation, and limited pollen and seed dispersal may collectively explain the high genetic differentiation among populations (Awad et al., 2014; Ge et al., 2005; Van Rossum et al., 2003). In populations of *C. tricoccon* where lizards are not present, introduced carnivorous mammals (e.g., pine martens and genets) may replace the native lizards as seed dispersers (Riera et al., 2002; Traveset, 1995b) although little is known about the implications for genetic diversity and population structure of this seed dispersal disruption. As pine martens and genets have extensive home ranges—up to 900 ha in the case of pine martens males (Clevenger, 1993)—higher gene flow may be expected in populations with carnivorous mammals as seed dispersers than those where lizards are still present, and therefore, low differentiation could be expected among these populations (Kirkpatrick & Barton, 1997). Surprisingly, no differences in genetic diversity and structure were found among populations with different dispersers in the Balearic Islands. These results could be explained by the habitat preferences of the introduced mammals. In a previous study, Celedón‐Neghme et al. (2013) showed that mammals replace lizards as seed dispersers only in populations occurring in the understory of holm oak forests. Therefore, gene flow mediated by introduced mammals is probably restricted to populations close to forested mountain areas instead of coastal maquis, the typical habitat of *C. tricoccon*. The low number of sampled populations occurring in holm oak forest (SA and LL) may account for the absence of significant differences in genetic diversity and population structure among populations with different dispersers. In other Tertiary relicts, where natural dispersers have disappeared, other species have occupied their role with important consequences for the species dispersion patterns (e.g., *Laurus nobilis*, Hampe, 2003).

The contrasting levels of genetic variation observed among populations can help identify valuable populations for conservation purposes. Assuming that genetic variation estimated with neutral molecular markers can reflect overall genetic diversity and be used as an indicator of variation in quantitative traits, several large populations with high genetic variation and different genetic makeup (e.g., FI, BL, DR) can be considered for further investigations into their conservation value. Accordingly, the low genetic variation found in some populations (e.g., FM, CG, GI, CR, CA, and ME) may compromise their long‐term viability. In particular, two populations (CA and ME) showed extremely low genetic variation, in terms of heterozygosity and polymorphism. Both populations are small and are geographically isolated in islands, where genetic drift can increase the fixation of deleterious alleles and outcrosses may be carried out between related parents. Therefore, reduced individual fitness and increased inbreeding depression can be expected in both populations (Kimura, Maruyama, & Crow, 1963; Lynch & Gabriel, 1990; Oakley & Winn, 2012). However, these populations are located at the dry edge of the species’ distribution range, and strong selection pressures related to water availability may be selecting drought‐adapted phenotypes, as has been suggested for marginal populations of other species (Kirkpatrick & Barton, 1997; Lesica & Allendorf, 1995), including Tertiary relicts such as *Ramonda myconi* (Muller et al., 1997). An experimental study with *C. tricoccon* adds support to this hypothesis, showing that dry‐edge populations (e.g., CA) may be locally adapted, as they exhibited multiple functional traits that favored drought tolerance (Lázaro‐Nogal et al., 2015). It is then likely that low genetic diversity and high divergence in these populations are explained by past strong directional selection combined with isolation‐driven low gene flow and strong genetic drift. Conservation of these *C. tricoccon* populations may be especially important in light of climate change as they may preserve genetic combinations that are important for adaptation to drier conditions (Hampe & Petit, 2005; Lesica & Allendorf, 1995).

In conclusion, our results highlight that, despite geographic isolation and important gene flow limitations related to pollen and seed dispersal mechanisms, population size can override, at least partially, the effects of geographical periphery. Integrative approaches taking into account different factors that determine genetic diversity and population structure, such as the position of a population in the species’ distribution range, size, the presence of effective seed and pollen dispersers, and the stability of local adaptations, are necessary to develop effective conservation strategies.

## Conflict of Interest

None declared.

## Author Contributions

A. L‐N., FV, and AT conceived the ideas; A L‐N collected the data; A L‐N, A G‐F, and SM analyzed the data; and A L‐N, A G‐F, and SM led the writing with contributions from all authors.

## References

[ece32940-bib-0001] Arnaud‐Haond, S. , Teixeira, S. , Massa, S. I. , Billot, C. , Saenger, P. , Coupland, G. , … Serrão, E. A. (2006). Genetic structure at range edge: Low diversity and high inbreeding in Southeast Asian mangrove (*Avicennia marina*) populations. Molecular Ecology, 15, 3515–3525. 17032254 10.1111/j.1365-294X.2006.02997.x

[ece32940-bib-0002] Avise, J. C. (2004). Molecular Markers, Natural History, and Evolution (Second Edition). Sinauer: Sunderland, MA.

[ece32940-bib-0003] Awad, L. , Fady, B. , Khater, C. , Roig, A. , & Cheddadi, R. (2014). Genetic structure and diversity of the endangered fir tree of Lebanon (*Abies cilicica* Carr.): Implications for conservation. PLoS One, 9, e90086. 24587219 10.1371/journal.pone.0090086 PMC3937446

[ece32940-bib-0004] Babweteera, F. , & Brown, N. (2009). Can remnant frugivore species effectively disperse tree seeds in secondary tropical rain forests? Biodiversity and Conservation, 18, 1611–1627.

[ece32940-bib-0005] Bauert, M. R. , Kalin, M. , Baltisberger, M. , & Edwards, P. J. (1998). No genetic variation detected within isolated relict populations of *Saxifraga cernua* in the Alps using RAPD markers. Molecular Ecology, 7, 1519–1527.

[ece32940-bib-0006] Brown, J. H. , Stevens, G. C. , & Kaufman, D. M. (1996). The geographic range: Size, shape, boundaries, and internal structure. Annual Review of Ecology and Systematics, 597–623.

[ece32940-bib-0007] Calviño‐Cancela, M. , Escudero, M. , Rodríguez‐Pérez, J. , Cano, E. , Vargas, P. , Velo‐Antón, G. , & Traveset, A. (2012). The role of seed dispersal, pollination and historical effects on genetic patterns of an insular plant that has lost its only seed disperser. Journal of Biogeography, 39, 1996–2006.

[ece32940-bib-0008] Celedón‐Neghme, C. , Traveset, A. , & Calviño‐Cancela, M. (2013). Contrasting patterns of seed dispersal between alien mammals and native lizards in a declining plant species. Plant Ecology, 214, 657–667.

[ece32940-bib-0009] Chapuis, M.‐P. , & Estoup, A. (2007). Microsatellite null alleles and estimation of population differentiation. Molecular Biology and Evolution, 24, 621–631. 17150975 10.1093/molbev/msl191

[ece32940-bib-0010] Christian, C. E. (2001). Consequences of a biological invasion reveal the importance of mutualism for plant communities. Nature, 413, 635–639. 11675787 10.1038/35098093

[ece32940-bib-0011] Chybicki, I. J. , & Burczyk, J. (2009). Simultaneous estimation of null alleles and inbreeding coefficients. Journal of Heredity, 100, 106–113. 18936113 10.1093/jhered/esn088

[ece32940-bib-0012] Clevenger, A. (1993). Pine marten (*Martes martes* L.) home ranges and activity patterns of the island of Minorca, Spain. Zeitschrift für Säugetierkunde, 58, 137–143.

[ece32940-bib-0013] Dobrowski, S. Z. (2011). A climatic basis for microrefugia: The influence of terrain on climate. Global Change Biology, 17, 1022–1035.

[ece32940-bib-0014] Dolan, R. W. (1994). Patterns of isozyme variation in relation to population size, isolation, and phytogeographic history in royal catchfly (*Silene regia*; Caryophyllaceae). American Journal of Botany, 965–972.

[ece32940-bib-0015] Dubreuil, M. , Riba, M. , & Mayol, M. (2008). Genetic structure and diversity in *Ramonda myconi* (Gesneriaceae): Effects of historical climate change on a preglacial relict species. American Journal of Botany, 95, 577–587. 21632384 10.3732/ajb.2007320

[ece32940-bib-0016] Durka, W. (1999). Genetic diversity in peripheral and subcentral populations of *Corrigiola litoralis* L. (Illecebraceae). Heredity, 83, 476–484. 10583550 10.1038/sj.hdy.6886000

[ece32940-bib-0017] Earl, D. A. , & vonHoldt, B. M. (2011). Structure Harvester: A website and program for visualizing STRUCTURE output and implementing the Evanno method. Conservation Genetics Resources, 4, 359–361.

[ece32940-bib-0018] Eckert, C. , Samis, K. , & Lougheed, S. (2008). Genetic variation across species’ geographical ranges: The central–marginal hypothesis and beyond. Molecular Ecology, 17, 1170–1188. 18302683 10.1111/j.1365-294X.2007.03659.x

[ece32940-bib-0019] Eckstein, R. L. , O'neill, R. , Danihelka, J. , Otte, A. , & Köhler, W. (2006). Genetic structure among and within peripheral and central populations of three endangered floodplain violets. Molecular Ecology, 15, 2367–2379. 16842412 10.1111/j.1365-294X.2006.02944.x

[ece32940-bib-0020] Ellstrand, N. C. , & Elam, D. R. (1993). Population genetic consequences of small population size: Implications for plant conservation. Annual Review of Ecology and Systematics, 24, 217–242.

[ece32940-bib-0021] Evanno, G. , Regnaut, S. , & Goudet, J. (2005). Detecting the number of clusters of individuals using the software structure: A simulation study. Molecular Ecology, 14, 2611–2620. 15969739 10.1111/j.1365-294X.2005.02553.x

[ece32940-bib-0023] Excoffier, L. , Smouse, P. E. , & Quattro, J. M. (1992). Analysis of molecular variance inferred from metric distances among DNA haplotypes: Application to human mitochondrial DNA restriction data. Genetics, 131, 479–491. 1644282 10.1093/genetics/131.2.479 PMC1205020

[ece32940-bib-0024] Falush, D. , Stephens, M. , & Pritchard, J. K. (2003). Inference of population structure using multilocus genotype data: Linked loci and correlated allele frequencies. Genetics, 164, 1567–1587. 12930761 10.1093/genetics/164.4.1567 PMC1462648

[ece32940-bib-0025] Feliner, G. N. (2014). Patterns and processes in plant phylogeography in the Mediterranean Basin. A review. Perspectives in Plant Ecology, Evolution and Systematics, 16, 265–278.

[ece32940-bib-0026] Frankham, R. (1996). Relationship of genetic variation to population size in wildlife. Conservation Biology, 10, 1500–1508.

[ece32940-bib-0027] Frankham, R. , Briscoe, D. A. , & Ballou, J. D. (2002). Introduction to conservation genetics. Cambridge University Press.

[ece32940-bib-0028] Gapare, W. J. , & Aitken, S. N. (2005). Strong spatial genetic structure in peripheral but not core populations of Sitka spruce [*Picea sitchensis* (Bong.) Carr.]. Molecular Ecology, 14, 2659–2667. 16029468 10.1111/j.1365-294X.2005.02633.x

[ece32940-bib-0029] Garcia‐Fernandez, A. , Lazaro‐Nogal, A. , Traveset, A. , & Valladares, F. (2012). Isolation and characterization of 10 microsatellite loci in *Cneorum tricoccon* (Cneoraceae), a Mediterranean relict plant. American Journal of Botany, 99, e307–e309. 22837406 10.3732/ajb.1100589

[ece32940-bib-0030] Garnatje, T. , Pérez‐Collazos, E. , Pellicer, J. , & Catalán, P. (2013). Balearic insular isolation and large continental spread framed the phylogeography of the western Mediterranean *Cheirolophus intybaceus* sl (Asteraceae). Plant Biology, 15, 166–175. 22759527 10.1111/j.1438-8677.2012.00632.x

[ece32940-bib-0031] Ge, X. J. , Zhou, X. L. , Li, Z. C. , Hsu, T. W. , Schaal, B. A. , & Chiang, T. Y. (2005). Low genetic diversity and significant population structuring in the relict *Amentotaxus argotaenia* complex (Taxaceae) based on ISSR fingerprinting. Journal of Plant Research, 118, 415–422. 16247653 10.1007/s10265-005-0235-1

[ece32940-bib-0032] González‐Varo, J. P. , López‐Bao, J. V. , & Guitián, J. (2013). Functional diversity among seed dispersal kernels generated by carnivorous mammals. Journal of Animal Ecology, 82, 562–571. 23228197 10.1111/1365-2656.12024

[ece32940-bib-0033] Goudet, J. (1995). FSTAT (Version 1.2): A computer program to calculate F‐statistics. Journal of Heredity, 86, 485–486.

[ece32940-bib-0034] Guillot, G. , Mortier, F. , & Estoup, A. (2005). GENELAND: A computer package for landscape genetics. Molecular Ecology Notes, 5, 712–715.

[ece32940-bib-0035] Hampe, A. (2003). Frugivory in European Laurel: How extinct seed dispersers have been substituted. Bird Study, 50, 280–284.

[ece32940-bib-0036] Hampe, A. , & Petit, R. J. (2005). Conserving biodiversity under climate change: The rear edge matters. Ecology Letters, 8, 461–467. 21352449 10.1111/j.1461-0248.2005.00739.x

[ece32940-bib-0037] Hampe, A. , & Petit, R. J. (2007). Ever deeper phylogeographies: Trees retain the genetic imprint of Tertiary plate tectonics. Molecular Ecology, 16, 5113–5114. 18092990 10.1111/j.1365-294X.2007.03604.x

[ece32940-bib-0038] Hamrick, J. , Murawski, D. A. , & Nason, J. D. (1993). The influence of seed dispersal mechanisms on the genetic structure of tropical tree populations In Fleming T. H., Alejandro Estrada, (Eds.), Frugivory and seed dispersal: Ecological and evolutionary aspects (pp. 281–297). Springer Netherlands.

[ece32940-bib-0039] Hewitt, G. M. (2011). Mediterranean peninsulas: The evolution of hotspots In Zachos Frank E., Jan Christian Habel, (Eds.), Biodiversity hotspots (pp. 123–147). Springer Berlin Heidelberg.

[ece32940-bib-0040] Hirao, A. S. (2010). Kinship between parents reduces offspring fitness in a natural population of *Rhododendron brachycarpum* . Annals of Botany, 105, 637–646. 20202970 10.1093/aob/mcq018 PMC2850802

[ece32940-bib-0041] Jacobsson, M. , & Rosenberg, N. A. (2007). CLUMPP: A cluster matching and permutation program for dealing with label switching and multimodality in analysis of population structure. Bioinformatics, 23, 1801–1806. 17485429 10.1093/bioinformatics/btm233

[ece32940-bib-0042] Jordano, P. , Forget, P.‐M. , Lambert, J. E. , Böhning‐Gaese, K. , Traveset, A. , & Wright, S. J. (2011). Frugivores and seed dispersal: Mechanisms and consequences for biodiversity of a key ecological interaction. Biology Letters, 7, 321–323. 21084336 10.1098/rsbl.2010.0986 PMC3097856

[ece32940-bib-0043] Kery, M. , Matthies, D. , & Spillmann, H. H. (2000). Reduced fecundity and offspring performance in small populations of the declining grassland plants *Primula veris* and *Gentiana lutea* . Journal of Ecology, 88, 17–30.

[ece32940-bib-0044] Kimura, M. , Maruyama, T. , & Crow, J. F. (1963). The mutation load in small populations. Genetics, 48, 1303. 14071753 10.1093/genetics/48.10.1303 PMC1210420

[ece32940-bib-0045] Kirkpatrick, M. , & Barton, N. H. (1997). Evolution of a species’ range. The American Naturalist, 150, 1–23. 10.1086/286054 18811273

[ece32940-bib-0046] Kluth, C. , & Bruelheide, H. (2005). Central and peripheral *Hornungia petraea* populations: Patterns and dynamics. Journal of Ecology, 93, 584–595.

[ece32940-bib-0047] Lammi, A. , Siikamäki, P. , & Mustajärvi, K. (1999). Genetic diversity, population size, and fitness in central and peripheral populations of a rare plant *Lychnis viscaria* . Conservation Biology, 13, 1069–1078.

[ece32940-bib-0048] Lawton, J. H. (1993). Range, population abundance and conservation. Trends in Ecology & Evolution, 8, 409–413. 21236213 10.1016/0169-5347(93)90043-O

[ece32940-bib-0049] Lázaro‐Nogal, A. , Forner, A. , Traveset, A. , & Valladares, F. (2013). Contrasting water strategies of two Mediterranean shrubs of limited distribution: Uncertain future under a drier climate. Tree Physiology, 33, 1284–1295. 24319030 10.1093/treephys/tpt103

[ece32940-bib-0050] Lázaro‐Nogal, A. , Matesanz, S. , Hallik, L. , Krasnova, A. , Traveset, A. , & Valladares, F. (2015). Population differentiation in a Mediterranean relict shrub: The potential role of local adaptation for coping with climate change. Oecologia, 180, 1075–1090. 26662734 10.1007/s00442-015-3514-0

[ece32940-bib-0051] Legendre, P. , & Legendre, L. (1998). Numerical ecology. Amsterdam, the Netherlands: Elsevier.

[ece32940-bib-0052] Lesica, P. , & Allendorf, F. W. (1995). When are peripheral populations valuable for conservation? Conservation Biology, 9, 753–760.

[ece32940-bib-0200] Lumaret, R. , Mir, C. , Michaud, H. , & Raynal, V. (2002). Phylogeographical variation of chloroplast DNA in holm oak (Quercus ilex L.). Molecular Ecology, 11, 2327–2336. 12406243 10.1046/j.1365-294x.2002.01611.x

[ece32940-bib-0053] Lynch, M. , & Gabriel, W. (1990). Mutation load and the survival of small populations. Evolution, 1725–1737. 28567811 10.1111/j.1558-5646.1990.tb05244.x

[ece32940-bib-0203] Müller, J. , Sprenger, N. , Bortlik, K. , Boller, T. , & Wiemken, A. (1997). Desiccation increases sucrose levels in Ramonda and Haberlea, two genera of resurrection plants in the Gesneriaceae. Physiologia Plantarum, 100, 153–158.

[ece32940-bib-0054] Myers, N. , Mittermeier, R. A. , Mittermeier, C. G. , Da Fonseca, G. A. , & Kent, J. (2000). Biodiversity hotspots for conservation priorities. Nature, 403, 853–858. 10706275 10.1038/35002501

[ece32940-bib-0055] Nybom, H. (2004). Comparison of different nuclear DNA markers for estimating intraspecific genetic diversity in plants. Molecular Ecology, 13, 1143–1155. 15078452 10.1111/j.1365-294X.2004.02141.x

[ece32940-bib-0056] Oakley, C. G. , & Winn, A. A. (2012). Effects of population size and isolation on heterosis, mean fitness, and inbreeding depression in a perennial plant. New Phytologist, 196, 261–270. 22816555 10.1111/j.1469-8137.2012.04240.x

[ece32940-bib-0057] Palamarev, E. (1989). Paleobotanical evidences of the Tertiary history and origin of the Mediterranean sclerophyll dendroflora. Plant Systematics and Evolution, 162, 93–107.

[ece32940-bib-0058] Peakall, R. , Ebert, D. , Scott, L. J. , Meagher, P. F. , & Offord, C. A. (2003). Comparative genetic study confirms exceptionally low genetic variation in the ancient and endangered relictual conifer, *Wollemia nobilis* (Araucariaceae). Molecular Ecology, 12, 2331–2343. 12919472 10.1046/j.1365-294x.2003.01926.x

[ece32940-bib-0059] Peakall, R. O. D. , & Smouse, P. E. (2006). GenAlEx 6: Genetic analysis in Excel. Population genetic software for teaching and research. Molecular Ecology Notes, 6, 288–295. 10.1093/bioinformatics/bts460 PMC3463245 22820204

[ece32940-bib-0060] Pérez‐Mellado, V. , Hernández‐Estévez, J. Á. , García‐Díez, T. , Terrassa, B. , Ramón, M. M. , Castro, J. , … Brown, R. (2008). Population density in *Podarcis lilfordi* (Squamata, Lacertidae), a lizard species endemic to small islets in the Balearic Islands (Spain). Amphibia‐Reptilia, 29, 49–60.

[ece32940-bib-0061] Pérez‐Méndez, N. , Jordano, P. , & Valido, A. (2015). Downsized mutualisms: Consequences of seed dispersers’ body‐size reduction for early plant recruitment. Perspectives in Plant Ecology, Evolution and Systematics, 17, 151–159.

[ece32940-bib-0062] Petit, R. J. , & Hampe, A. (2006). Some evolutionary consequences of being a tree. Annual Review of Ecology, Evolution and Systematics, 37, 187–214.

[ece32940-bib-0063] Pironon, S. , Papuga, G. , Villellas, J. , Angert, A. L. , García, M. B. , & Thompson, J. D. (2016). Geographic variation in genetic and demographic performance: new insights from an old biogeographical paradigm. Biological Reviews, doi:10.1111/brv.12313 10.1111/brv.12313 27891813

[ece32940-bib-0064] Pritchard, J. K. , Stephens, M. , & Donnelly, P. (2000). Inference of population structure using multilocus genotype data. Genetics, 155, 945–959. 10835412 10.1093/genetics/155.2.945 PMC1461096

[ece32940-bib-0065] Pulliam, H. R. (2000). On the relationship between niche and distribution. Ecology Letters, 3, 349–361.

[ece32940-bib-0066] Raven, P. H. (1973). The evolution of Mediterranean floras In Di Castri F., & Mooney H. A. (Eds.), Mediterranean type ecosystems: Origin and structure (pp. 213–224). Berlin, Heidelberg, Germany: Springer Berlin Heidelberg.

[ece32940-bib-0067] Riera, N. , Traveset, A. , & García, O. (2002). Breakage of mutualisms by exotic species: The case of *Cneorum tricoccon* L. in the Balearic Islands (Western Mediterranean Sea). Journal of Biogeography, 29, 713–719.

[ece32940-bib-0068] Rodríguez‐Sánchez, F. , Guzman, B. , Valido, A. , Vargas, P. , & Arroyo, J. (2009). Late Neogene history of the laurel tree (*Laurus L*., Lauraceae) based on phylogeographical analyses of Mediterranean and Macaronesian populations. Journal of Biogeography, 36, 1270–1281.

[ece32940-bib-0069] Rodríguez‐Sánchez, F. , Perez‐Barrales, R. , Ojeda, F. , Vargas, P. , & Arroyo, J. (2008). The Strait of Gibraltar as a melting pot for plant biodiversity. Quaternary Science Reviews, 27, 2100–2117.

[ece32940-bib-0070] Rosenberg, N. A. (2004). Distruct: A program for the graphical display of population structure. Molecular Ecology Notes, 4, 137–138.

[ece32940-bib-0071] Rosenberg, M. S. , & Anderson, C. D. (2011). PASSaGE: Pattern analysis, spatial statistics and geographic exegesis. Version 2. Methods in Ecology and Evolution, 2, 229–232.

[ece32940-bib-0072] Rousset, F. (2008). genepop'007: A complete re‐implementation of the genepop software for Windows and Linux. Molecular Ecology Resources, 8, 103–106. 21585727 10.1111/j.1471-8286.2007.01931.x

[ece32940-bib-0073] Sagarin, R. D. , & Gaines, S. D. (2002). The ‘abundant centre’ distribution: To what extent is it a biogeographical rule? Ecology Letters, 5, 137–147.

[ece32940-bib-0074] Schaal, B. A. , & Leverich, W. J. (1996). Molecular variation in isolated plant populations. Plant Species Biology, 11, 33–40.

[ece32940-bib-0075] Schuelke, M. (2000). An economic method for the fluorescent labeling of PCR fragments. Nature Biotechnology, 18, 233–234. 10.1038/72708 10657137

[ece32940-bib-0076] Traveset, A. (1995a). Reproductive ecology of *Cneorum tricoccon* L. (Cneoraceae) in the Balearic Islands. Botanical Journal of the Linnean Society, 117, 221–232.

[ece32940-bib-0077] Traveset, A. (1995b). Seed dispersal of *Cneorum tricoccon* L. (Cneoraceae) by lizards and mammals in the Balearic Islands. Acta Ecologica, 171, 178.

[ece32940-bib-0078] Traveset, A. , Gonzalez‐Varo, J. P. , & Valido, A. (2012). Long‐term demographic consequences of a seed dispersal disruption. Proceedings of the Royal Society of London B: Biological Sciences, rspb20120535. 10.1098/rspb.2012.0535 PMC3385730 22628466

[ece32940-bib-0079] Traveset, A. , Quintana, J. , & Alcover, J. A. (2005). Fossil seeds from the Pliocene of Menorca and Eivissa (Balearic Islands, Western Mediterranean). Endins, 27, 205–210.

[ece32940-bib-0080] Van Rossum, F. , Vekemans, X. , Gratia, E. , & Meerts, P. (2003). A comparative study of allozyme variation of peripheral and central populations of *Silene nutans* L. (Caryophyllaceae) from Western Europe: Implications for conservation. Plant Systematics and Evolution, 242, 49–61.

[ece32940-bib-0081] Vanden‐Broeck, A. , Gruwez, R. , Cox, K. , Adriaenssens, S. , Michalczyk, I. M. , & Verheyen, K. (2011). Genetic structure and seed‐mediated dispersal rates of an endangered shrub in a fragmented landscape: A case study for *Juniperus communis* in northwestern Europe. BMC Genetics, 12, 1. 21859457 10.1186/1471-2156-12-73 PMC3176195

[ece32940-bib-0082] Vesica, P. , Tuccimei, P. , Turi, B. , Fornós, J. , Ginés, A. , & Ginés, J. (2000). Late Pleistocene paleoclimates and sea‐level change in the Mediterranean as inferred from stable isotope and U‐series studies of overgrowths on speleothems, Mallorca, Spain. Quaternary Science Reviews, 19, 865–879.

[ece32940-bib-0083] Weir, B. S. , & Cockerman, C. C. (1984). Estimating F‐statistics for the analysis of population structure. Evolution, 38, 1358–1370. 28563791 10.1111/j.1558-5646.1984.tb05657.x

