# Supplementary material for: Population size, center–periphery, and seed dispersers’ effects on the genetic diversity and population structure of the Mediterranean relict shrub Cneorum tricoccon
Source: Ecol Evol. 2017 Aug 5;7(18):7231–42. doi: 10.1002/ece3.2940 (PMC5606867; doi:10.1002/ece3.2940)
Supplement: Supplementary file 1 [file ECE3-7-7231-s001.docx]

**SUPPORTING INFORMATION**

|  | **SA** | **LL** | **BL** | **CO** | **TG** | **BB** | **CA** | **CC** | **DR** | **ME** |  | **GI** | **CR** | **FI** | **MO** | **MA** |
| --- | --- | --- | --- | --- | --- | --- | --- | --- | --- | --- | --- | --- | --- | --- | --- | --- |
| **LL** | 0.2769 |  |  |  |  |  |  |  |  |  |  |  |  |  |  |  |
| **BL** | 0.3419 | 0.2774 |  |  |  |  |  |  |  |  |  |  |  |  |  |  |
| **CO** | 0.3440 | 0.3070 | 0.1997 |  |  |  |  |  |  |  |  |  |  |  |  |  |
| **TG** | 0.3420 | 0.3426 | 0.2351 | 0.2520 |  |  |  |  |  |  |  |  |  |  |  |  |
| **BB** | 0.2905 | 0.2871 | 0.1798 | 0.1662 | 0.1993 |  |  |  |  |  |  |  |  |  |  |  |
| **CA** | 0.6945 | 0.6666 | 0.6896 | 0.7245 | 0.7414 | 0.7530 |  |  |  |  |  |  |  |  |  |  |
| **CC** | 0.4454 | 0.3962 | 0.1891 | 0.2826 | 0.3256 | 0.2877 | 0.7600 |  |  |  |  |  |  |  |  |  |
| **DR** | 0.4337 | 0.4599 | 0.4495 | 0.4416 | 0.4758 | 0.4381 | 0.7642 | 0.4934 |  |  |  |  |  |  |  |  |
| **ME** | 0.6488 | 0.6490 | 0.6812 | 0.5690 | 0.6654 | 0.6509 | **0.9764** | 0.7281 | 0.7340 |  |  |  |  |  |  |  |
| **GI** | 0.5776 | 0.4768 | 0.5568 | 0.4861 | 0.5647 | 0.5439 | 0.9087 | 0.6398 | 0.6621 | 0.8432 |  |  |  |  |  |  |
| **CR** | 0.5501 | 0.5131 | 0.5786 | 0.4989 | 0.5141 | 0.5181 | 0.9186 | 0.6318 | 0.6470 | 0.8423 |  | 0.7369 |  |  |  |  |
| **FI** | 0.3572 | 0.3060 | 0.3148 | 0.3788 | 0.3564 | 0.3652 | 0.6854 | 0.3902 | 0.4140 | 0.6775 |  | 0.5533 | 0.5618 |  |  |  |
| **MO** | 0.5627 | 0.5282 | 0.4714 | 0.4733 | 0.5438 | 0.5298 | 0.8753 | 0.5492 | 0.6257 | 0.7984 |  | 0.7616 | 0.7321 | 0.4981 |  |  |
| **MA** | 0.5886 | 0.5917 | 0.4996 | 0.5263 | 0.4768 | 0.5372 | 0.9351 | 0.5726 | 0.7175 | 0.9127 |  | 0.8045 | 0.8107 | 0.6320 | 0.7771 |  |
| **CG** | 0.5805 | 0.5864 | 0.5138 | 0.5343 | 0.4647 | 0.5351 | 0.9341 | 0.5824 | 0.7134 | 0.9117 |  | 0.8024 | 0.8051 | 0.6280 | 0.7801 | **0.1530** |
|  |  |  |  | Central |  |  |  |  |  |  |  |  |  | Peripheral | |  |

**Table S1.** Pairwise *F*_ST_ values for all population comparisons. All values are significant (*P* ≤0.001). The highest and lowest *F*_ST_ values are in bold.

**Figure S1.** Population structure inferred by Bayesian cluster analyses (STRUCTURE) in the 16 *C. tricoccon* sampled populations. Results for *K=* 6 are shown. Letter codes correspond to the population listed in Table 1.


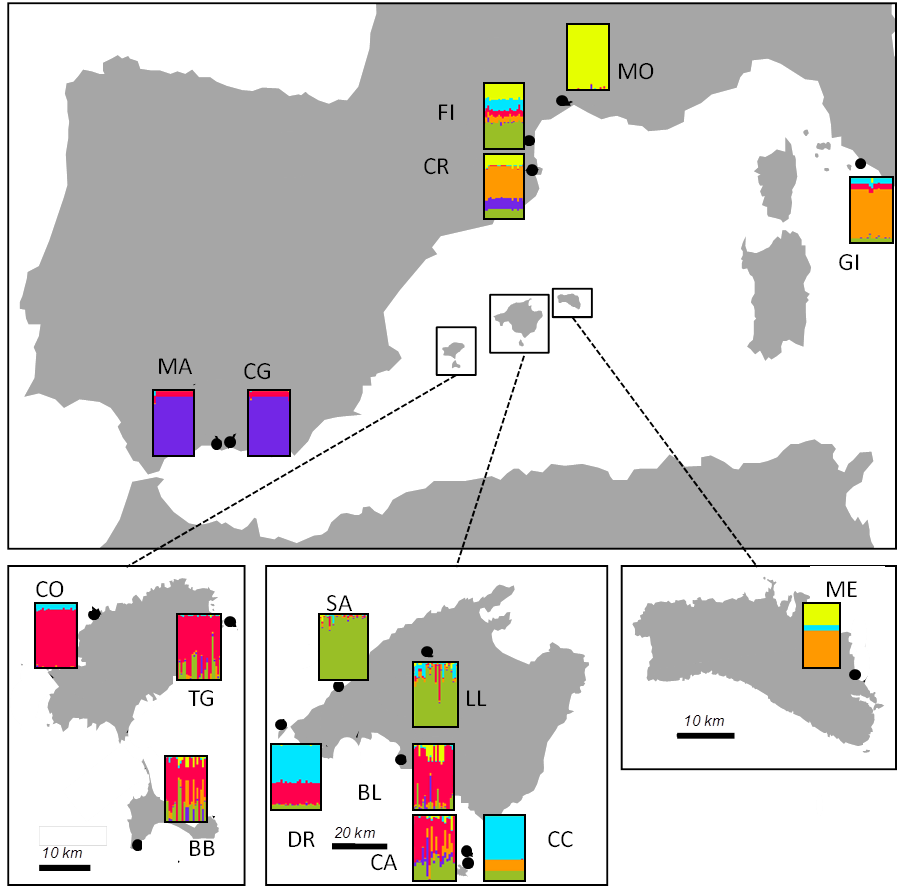


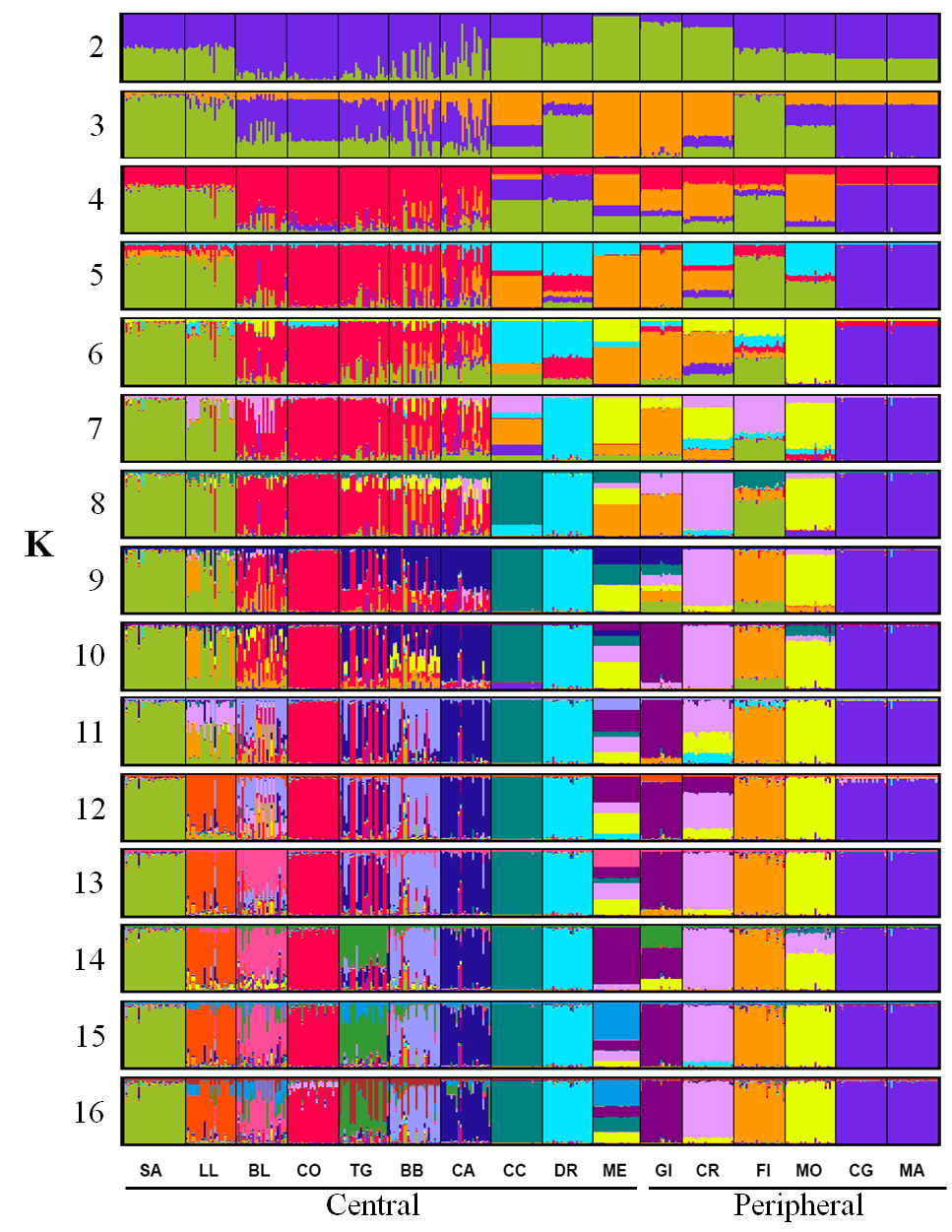
**Figure S2.** Population structure inferred by Bayesian cluster analyses (STRUCTURE for 399 *Cneorum tricoccon* individuals from 16 central and peripheral populations. Results for *K* (number of clusters) ranging from 2 to 16. Each individual (grouped by population) is represented by a vertical bar. Letter codes correspond to the population listed in Table 1.

**Figure S3.** Principal Coordinate Analysis (PCoA) for 16 populations of *Cneorum tricoccon*. The first and second access account for ≈40% of the variation. The colour codes for each population reflects the clustering performed by Structure, with *K*=6. Letter codes correspond to the population listed in Table 1.

**
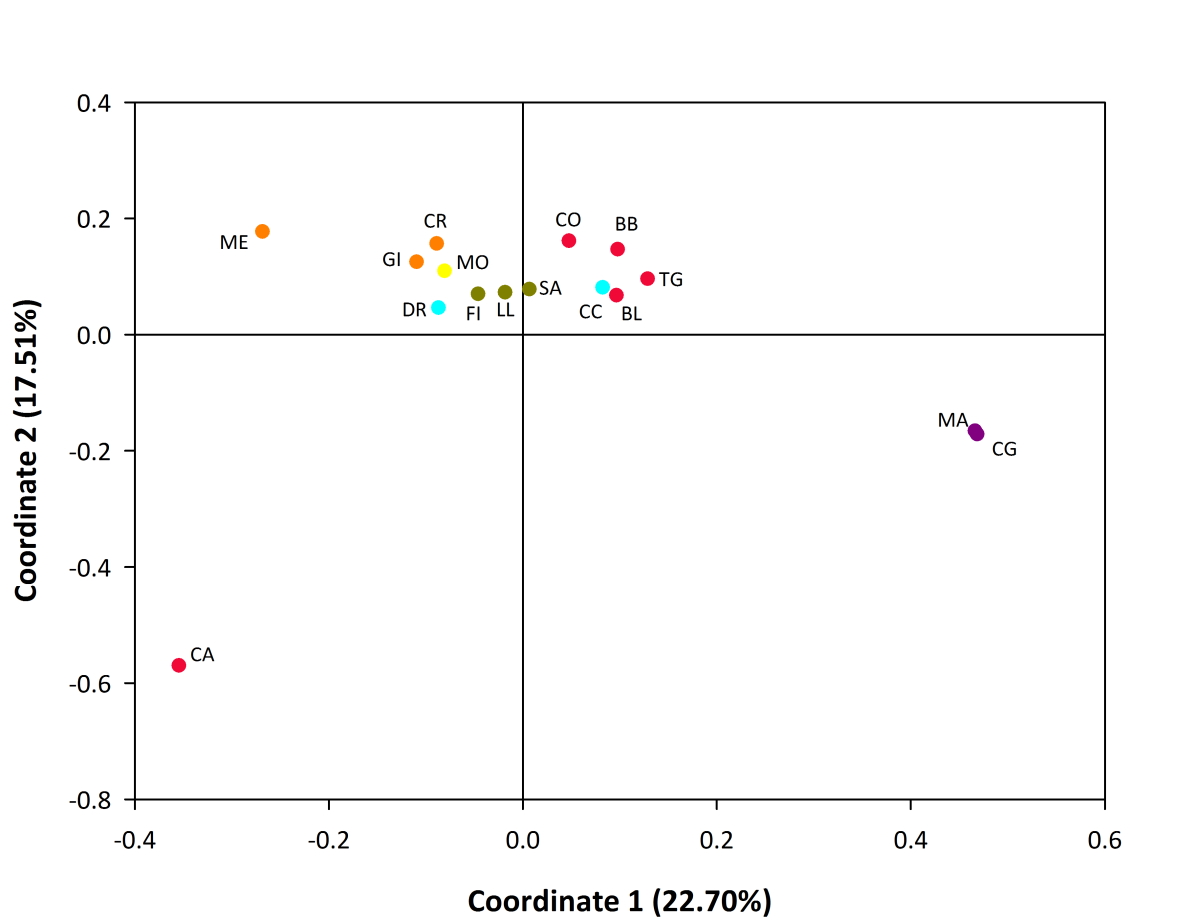
**
